# Ultraviolet (UV) radiation: a double-edged sword in cancer development and therapy

**DOI:** 10.1186/s43556-024-00209-8

**Published:** 2024-10-17

**Authors:** Zhen-wei Yu, Min Zheng, Hua-yang Fan, Xin-hua Liang, Ya-ling Tang

**Affiliations:** 1 State Key Laboratory of Oral Diseases & National Clinical Research Center for Oral Diseases & Department of Oral and Maxillofacial Surgery, West China Hospital of Stomatology, Sichuan University, No.14, Sec.3, Renminnan Road, Chengdu, Sichuan 610041 People’s Republic of China; 2 Department of Stomatology, Zhoushan Hospital, Wenzhou Medical University, Zhoushan, Zhejiang China; 3 State Key Laboratory of Oral Diseases & National Clinical Research Center for Oral Diseases & Department of Oral Pathology, West China Hospital of Stomatology, Sichuan University, No.14, Sec.3, Renminnan Road, Chengdu, Sichuan 610041 People’s Republic of China

**Keywords:** Ultraviolet (UV) light, Ultraviolet (UV) radiation, Cancer, Carcinogenesis, Nanoparticles, Cancer therapy

## Abstract

It has long been widely acknowledged that ultraviolet (UV) light is an environment risk factor that can lead to cancer, particularly skin cancer. However, it is worth noting that UV radiation holds potential for cancer treatment as a relatively high-energy electromagnetic wave. With the help of nanomaterials, the role of UV radiation has caught increasing attention in cancer treatment. In this review, we briefly summarized types of UV-induced cancers, including malignant melanoma, squamous cell carcinoma, basal cell carcinoma, Merkel cell carcinoma. Importantly, we discussed the primary mechanisms underlying UV carcinogenesis, including mutations by DNA damage, immunosuppression, inflammation and epigenetic alterations. Historically limited by its shallow penetration depth, the introduction of nanomaterials has dramatically transformed the utilization of UV light in cancer treatment. The direct effect of UV light itself generally leads to the suppression of cancer cell growth and the initiation of apoptosis and ferroptosis. It can also be utilized to activate photosensitizers for reactive oxygen species (ROS) production, sensitize radiotherapy and achieve controlled drug release. Finally, we comprehensively weigh the significant risks and limitations associated with the therapeutic use of UV radiation. And the contradictory effect of UV exposure in promoting and inhibiting tumor has been discussed. This review provides clues for potential clinical therapy as well as future study directions in the UV radiation field. The precise delivery and control of UV light or nanomaterials and the wavelength as well as dose effects of UV light are needed for a thorough understanding of UV radiation.

## Introduction

**Fig. 1:**
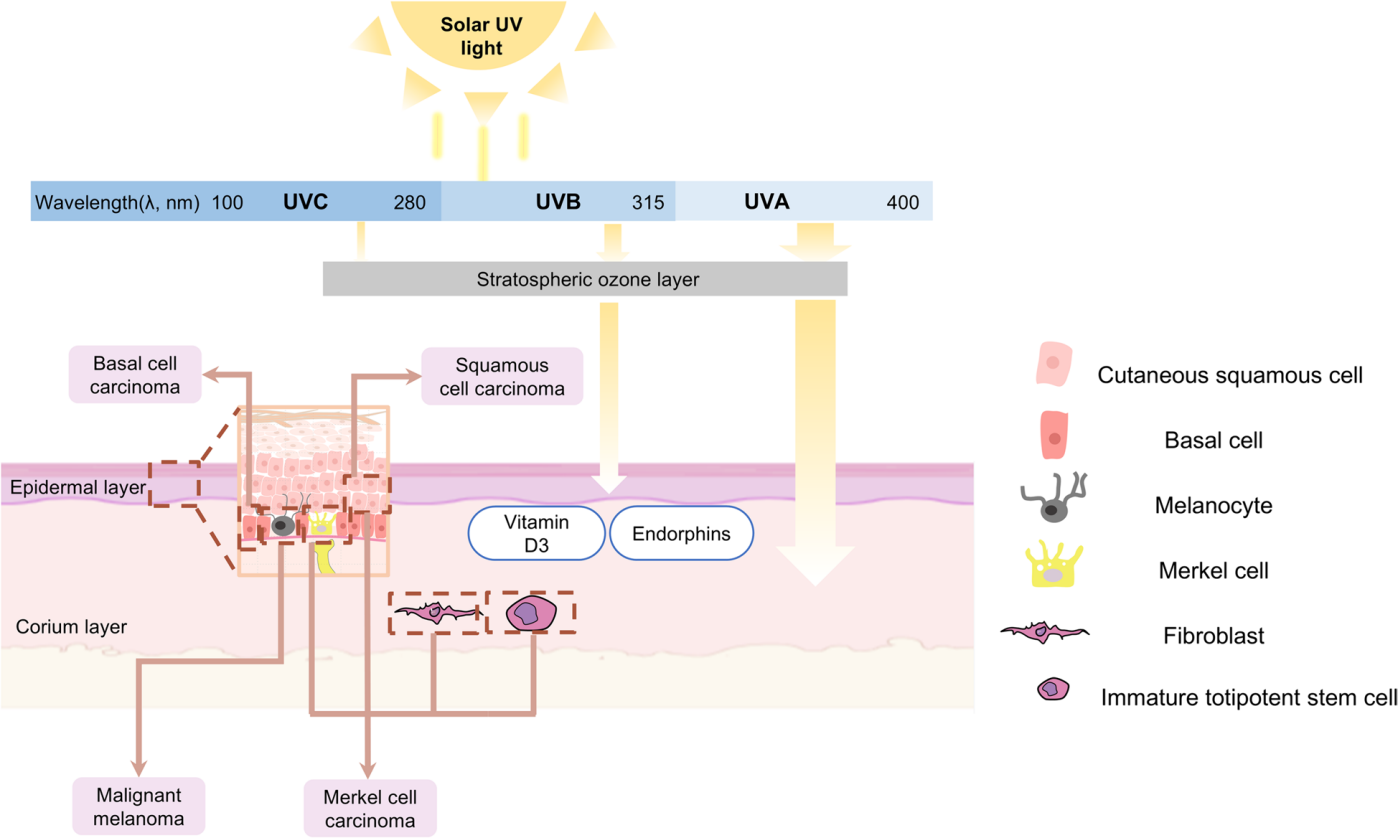
The relationship between UV and skin. The three types of UV rays, primarily UVA and UVB, penetrate to different depths in the skin tissue. UVA can reach the dermis layer of the skin, while UVB is limited to the epidermis and the uppermost layer of the dermis. In normal, UVB boosts vitamin D3 and endorphin synthesis. However, excessive UV exposure can lead to the formation of cancers including melanoma, basal cell carcinoma, squamous cell carcinoma, and Meckel cell carcinoma. The box shows the different cell sources of these cancers

As the information from the World Health Organization (WHO), close to 20 million new cases of cancer occurred in the year 2022 paralleled by 9.7 million deaths due to it [1]. In recent years, the incidence of skin cancer, especially squamous cell carcinoma, has been reported to be increased significantly. Due to long-term exposure to the external environment, the skin is the most vulnerable organ of the human body after ultraviolet (UV) irradiation and it is reported that 90% of skin cancers are related to UV radiation damage. The significance of UV light or radiation as a pivotal contributor to cancer development is extensively acknowledged [2–4]. UV light is an electromagnetic wave separated as long-wave UVA (315–400 nm), mid-wave UVB (280–315 nm), and short-wave UVC (100–280 nm) [5]. The majority of the UV radiation reaching earth's surface is UVA, with a smaller proportion being UVB. In contrast, UVC is predominantly absorbed by the ozone layer located in the stratosphere (Fig. 1). UVA, accounting for 90%-95% of the total UV, has a high penetration ability. The radiation exposure extends into the papillary layer of the dermis, influencing cellular elements within the dermis and the subcutaneous region, such as fibroblasts, endothelial cells of blood vessels, and Langerhans cells, and stimulates the activity of matrix metalloproteinases (MMPs), leading to the breakdown of collagen and elastin fibers, which causes visible photoaging damage such as skin relaxation, sagging, and abnormal increase of wrinkles [6]. The content of UVB is low (5%~10% of the total UV) but it has high energy, which is mainly absorbed by the epidermis. The effects of UVB includes stratum corneum thickening, epidermal hyperplasia, melanocytes proliferation, induced DNA mutations and chromosomal variants in keratinocytes, which leads to cell carcinogenesis [7]. The shorter the wavelength of UV light, the higher its energy content. Usually, the production of vitamin D and endorphins could be stimulated mainly by UVB light [8, 9]. Nonetheless, prolonged and excessive exposure to UV light can have adverse effects on the skin, such as atrophy, dyspigmentation, wrinkles formation, and an elevated risk of malignant transformation. Previous research often portrays UV light as a harmful factor and therefore has proposed prevention and treatment strategies based on current understanding [2, 10–13].

However, every coin has two sides. Sunlight has been applied to treat a variety of diseases since BC. Finsen won the Nobel Prize in Physiology and Medicine in 1903 for his success in treating disease with concentrated light irradiation, especially in treating cutaneous tuberculosis. In the 20th century, Goeckerman began to use high pressure mercury lamp combined with external application of coal tar to treat psoriasis and achieved good results, which was the beginning of modern phototherapy of UV radiation and also intriguingly presents a potential for its application in the treatment of cancer directly or indirectly [14].

Although the therapeutic use of UV light on cancer was often limited by its poor penetration depth in the past, these drawbacks are largely circumvented for the development of nanomaterials at present [15–18]. UV light itself usually functions by inhibiting cancer cell proliferation, inducing their apoptosis or ferroptosis, enhancing radiotherapy sensitivity, triggering chemical reactions to enable controlled-drug release and facilitating the creation of reactive oxygen species (ROS) in photodynamic therapy (PDT) [19–23]. On the other hand, UV light can serve as a stimulant to activate certain particles or molecules that produce novel effects and destroy cancer cells [24–28]. With the widespread appearance and promotion of nanomaterials, the role of UV radiation has caught increasing attention in cancer treatment.

In this review, we briefly introduce UV-induced cancers and mechanisms of carcinogenesis. Furthermore, we focus on summarizing the current applications of UV radiation, both in itself and as a stimulant in the treatment of cancer. In addition, given UV radiation's well-established role as a carcinogen, we comprehensively weigh the significant risks and limitations associated with the therapeutic use of UV radiation including potential long-term effects, the challenges of targeted delivery, and the risk of inducing secondary cancers. And the contradictory effect of UV exposure in promoting and inhibiting cancer has been discussed. It will assist researchers in understanding the dual relationship between UV radiation and cancer and developing more favorable cancer treatment strategies based on UV radiation.

## Photocarcinogenesis: UV-induced cancers

A growing amount of research indicates that UV radiation exposure is linked to the emergence and growth of cancers in several body areas [4, 29–34]. Specially, skin cancer, encompassing both malignant melanoma and non-melanoma skin cancers (NMSCs), exhibits the strongest correlation with UV exposure (Fig. 1). Conditions are predominantly observed in individuals with lighter skin pigmentation and are especially prevalent in regions that receive frequent solar exposure, such as the head and neck [29, 35–38].

### Malignant melanoma

Malignant melanoma is famous for its challenging responsiveness to therapeutic approaches and its propensity for metastatic dissemination with the ability to occur in different parts of the body [39–41]. Superficial spreading melanoma (SSM), a melanoma subtype closely linked to cumulative solar damage, initially presents its pathogenic effects within the superficial epidermal layers. Nevertheless, this neoplasm is distinguished by its rapid propensity to invade the dermis, subsequently infiltrates the lymphatic pathways and extends to distant organs [41, 42]. A positive association has been consistently observed between annual UV exposure and the incidence of melanoma. The substantial surge in melanoma cases affecting the trunk and upper limbs is likely a consequence of the growing prevalence of desultory, high-intensity recreational UV radiation exposure, such as sunbathing and the use of indoor tanning facilities [43].

### Non-melanoma skin cancers

In the realm of NMSCs, squamous cell carcinoma (SCC) and basal cell carcinoma (BCC) reign supreme, accounting for approximately 20 to 25 % and 75 to 80 % of cases respectively. Among the remaining cases, Merkel cell carcinoma (MCC) constitutes a rare yet significant proportion [44]. Despite the higher incidence rates of BCC and SCC, their impact on life-threatening potential is significantly less when compared to malignant melanoma. Investigations have revealed a significant link correlating the risk factors of BCC and SCC with various parameters of UV exposure including dosage, intensity, and the specific mode of radiation, underscoring the critical role of UV in the etiology of these malignancies. Furthermore, individual factors such as fair skin pigmentation often amplify one's susceptibility to UV radiation, which contribute to an individual's vulnerability to NMSCs [45, 46].

### Squamous cell carcinoma

SCC arises from the squamous cells that form the outermost layers of the epidermis namely the epidermis's protective shell [45]. UVB radiation stands out as the paramount contributor to the etiology of cutaneous squamous cell carcinoma (cSCC) among environmental factors and accumulated total solar UV radiation exposure has been positively associated with an elevated incidence of cSCC, which has a significant impact on the molecular pathogenesis [47, 48]. Additionally, UV could perturb extracellular matrix (ECM) components such as metalloproteinases and collagen to promote the progression of cSCC [49]. It also synergizes with certain cutaneous human papillomavirus (HPV) types potentiating UVR-mediated damage at an initial stage of cSCC carcinogenesis [50]. Exposure to UV can cause mutations in individual keratinocytes and give these cells a survival advantage. With environmental selection, such keratinocytes gradually form many subclones in clinically normal appearing skin and can gradually develop polyclonal cSCC as genetic or epigenetic changes accumulate [51, 52]. Meanwhile, the progression of Actinic Keratosis (AK) is predominantly attributed to UV exposure, which is characterized by the presence of atypical cellular alterations within the epidermal keratinocytes and considered as a significant precursor lesion for SCC [53].

### Basal cell carcinoma

BCC is the most common type of skin cancer in humans, originating from the cells that constitute the basal layer of the epidermis with clinical presentation associated with its subtype. UV radiation is primarily responsible for causing BCC, and the time gap between UV exposure and the onset of BCC can span several decades. Cumulative exposure to environmental UV radiation is associated with a rise in both the relative and absolute risks of BCC, peaking in the head and neck region, whereas intermittent intense UV exposure during childhood and adolescence such as outdoor activities is correlated with a significant increase in subsequent BCC risk in this age group [54]. Comparing those with little occupational UV exposure to those with high levels, the latter group had a significantly higher BCC risk [55].

### Merkel cell carcinoma

Merkel cell carcinoma (MCC) emerges as a neuroendocrine skin cancer characterized by its rarity and preferential occurrence on the head and neck with high aggressiveness and mortality as well as a worse prognosis [56]. Recent evidence suggests that MCC is more likely to originate from dermal fibroblasts, epidermal keratinocytes, or immature totipotent stem cells that undergo neuroendocrine differentiation during the process of malignant transformation [57, 58]. UV light and human Merkel cell polyomavirus (MCPyV) are thought to be the main cause. Chronic exposure to sunlight can result in two main biological effects [59]. One is UV‑mediated DNA damage with mutational signatures that are characteristic of UV exposure, which is mainly found in MCPyV^−^ MCCs in countries with high UV exposure. UV radiation specifically promotes the development of MCC by activating the Wnt signaling pathway and inducing the expression of MMPs. Furthermore, it contributes to the accumulation of mutations in genes critical for cell cycle regulation and neuroendocrine differentiation, such as *TP53*, *RB1*, *AKT1*, *BCL-2*, and *c-MYC*, thereby facilitating the promotion of carcinogenesis in MCC [57, 58]. On the other hand, UV radiation is implicated in the enhancement of MCPyV replication and its subsequent integration into the host genome through its induction of immunosuppression, thereby contributing to the pathogenesis of MCC [60].

## Mechanism of carcinogenesis by UV

**Fig. 2:**
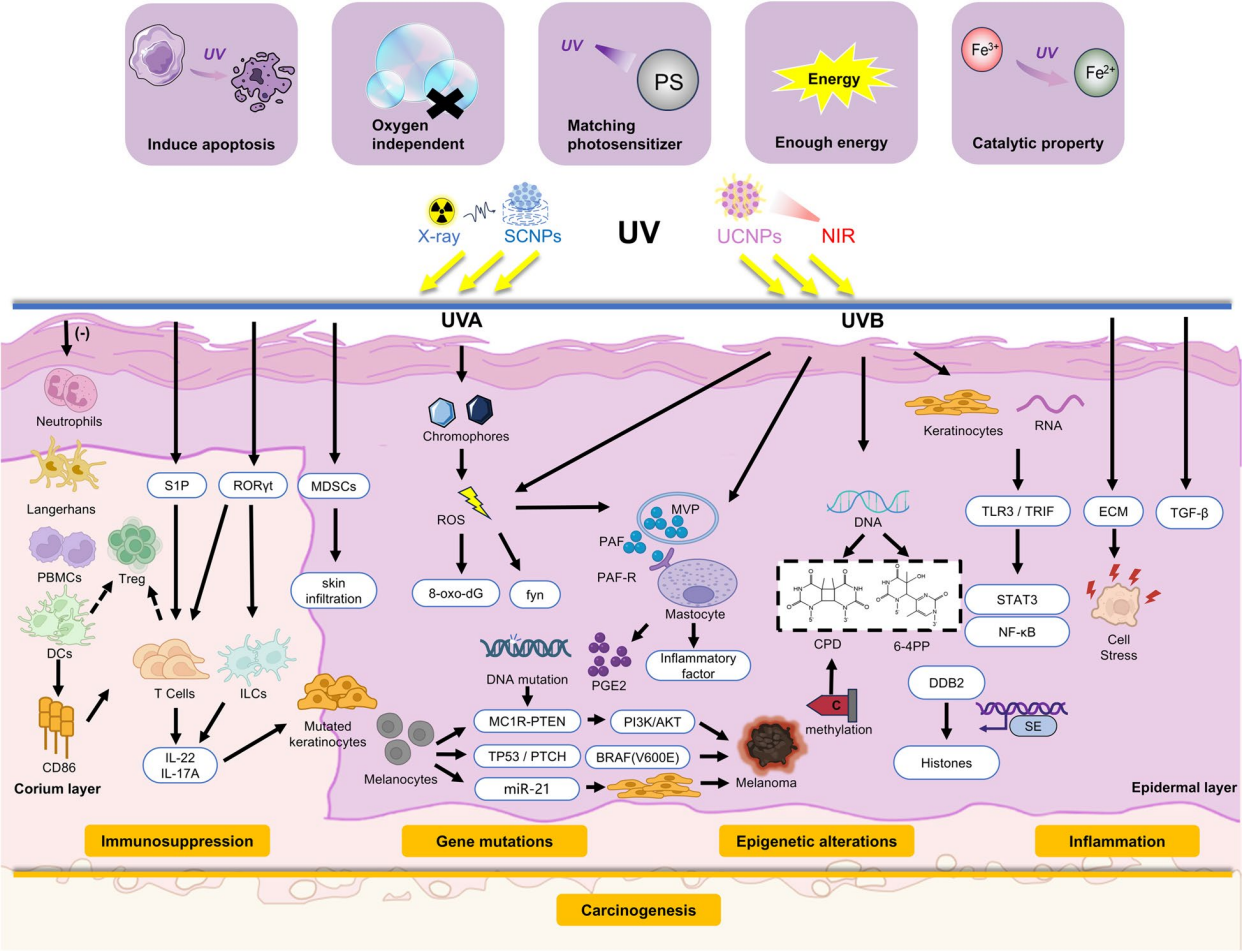
Summary of the dual relationship between UV and tumor. Genetic mutations, immunosuppression, inflammation, epigenetic alterations, and their interplays are the main mechanisms and pathways for UV-induced cancer, which demonstrates role of UV in carcinogenesis. In addition, properties of UV light including inducing apoptosis, oxygen independent, matching photosensitizer, enough energy, catalytic property serve as the basis for its role in tumor therapy, which indicates its potential for tumor treatment

UV has been increasingly studied for its carcinogenic mechanisms because of its widespread impact and distribution. UV radiation has been shown to directly instigate DNA damage and indirectly produce ROS, which subsequently result in mutations. Concurrently, UV radiation possesses the capacity to modify the functions of immune cells through the mediation of inflammatory factors and pro-inflammatory agents. Epigenetic changes and inflammation are also involved in the malignant transformation process. More complicated, these primary mechanisms are known to interplay with one another (Fig. 2). This intricate interplay between UV radiation and skin biology emphasizes the crucial need for a deeper exploration of these mechanisms to guide the development of potent preventative and therapeutic measures.

### Mutations: directly or indirectly DNA damage

#### Direct DNA damage

**Fig. 3:**
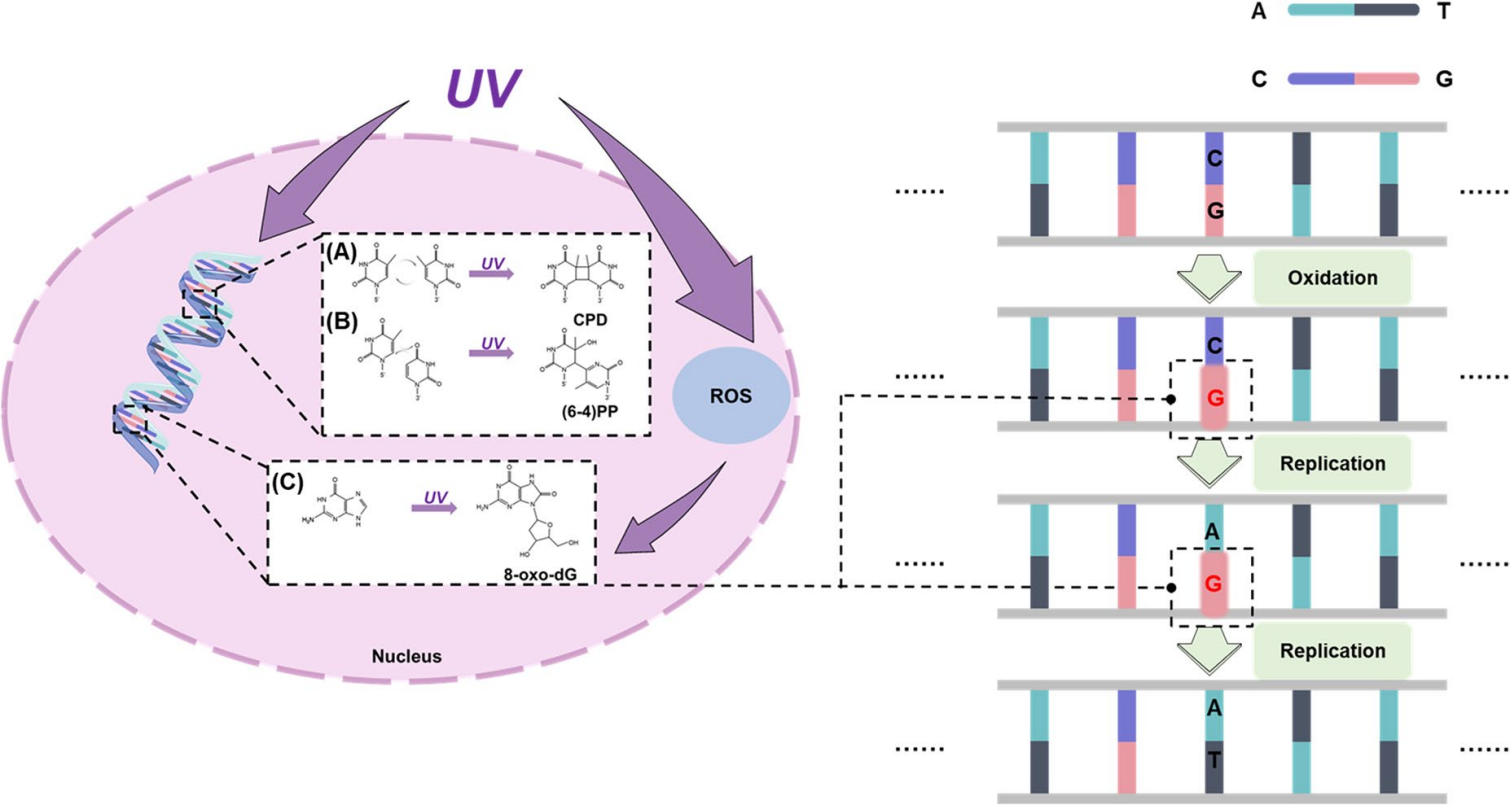
UV-photoproducts and base mismatch. UV directly, or indirectly through ROS lead to the formation of DNA photoproducts. For example, based on thymine and guanine, DNA photoproducts that may lead to the formation of mutations mainly are (**A**) cyclobutane pyrimidine dimers (CPDs), (**B**) pyrimidine (6–4) pyrimidone photoproducts (6-4PPs), and (**C**) 8-oxo-7,8-dihydro-2'-deoxyguanosine (8-oxo-dG). Specially, 8-oxo-dG can mismatch with adenine during DNA replication and result in G > T transversion during subsequent replication

The bulk of direct DNA damage stem predominantly from UVB radiation, which produces multiple types of DNA damage such as DNA strand breaks, DNA adducts, DNA–protein crosslinks [61, 62]. Among the photoproducts after UV exposure, cyclobutane pyrimidine dimer (CPDs) and pyrimidine (6-4) pyrimidone photoproducts (6-4PPs) are the two most important (Fig. 3). The former arises from a [2+2] cycloaddition of the C5-C6 double bonds of adjacent pyrimidine bases and the latter arises from a [2+2] cycloaddition involving the C5-C6 double bond of the 5 '-end pyrimidine and the C4 carbonyl group of the 3′-end thymine [63]. However, the greatest interest with respect to UV light carcinogenesis has been in CPDs, which are the major DNA photoproducts formed following UV light and repaired in human skin primarily by nucleotide excision repair (NER) [62, 64–66]. Tommasi *et al.* has found methylation of DNA bases significantly increases their vulnerability to UV-induced lesions. When cells are irradiated with natural sunlight, CPDs formation significantly increased due to the presence of 5-methylcytosine bases [67]. It is reported that Poly(ADP-ribosyl)ation (PARylation) can occur in DDB1 through PARP1 thereby inhibiting DNA damage repair when DNA is damaged, which may be an important mechanism of DNA repair dysregulation under UV exposure [68].

#### Indirect DNA damage—Oxidative stress

Meanwhile, both types of UV radiation are considered to elicit epidermal cells damage and eventually induce cancer for genome damage caused by oxidative stress (the primary mechanism for UVA) [64, 69–71]. Chromophores resident within skin cells including DNA, tryptophan, and urocanic acid, act as receptors for UV photons and absorb them to generate ROS. After that, cell signaling pathways are activated through a key mediator namely fyn, a key constituent of the Src family of protein tyrosine kinases with pivotal role in modulation of signal transduction pathways [72]. These ROS possess the capacity to inflict oxidative damage upon proteins and DNA, thereby predisposing to base mispairing and the induction of genetic mutations. Furthermore, these ROS can significantly augment the glucose metabolism in melanoma cells [73, 74]. A notable consequence of DNA's exposure to ROS is the formation of oxidized guanosine namely 8-oxo-7,8-dihydro-2'-deoxyguanosine (8-oxo-dG) (Fig. 3). It is recognized by 8-oxoguanine DNA glycosylase (OGG1), repaired by base excision repair (BER) and generated after UV exposure, which will pair with adenine during DNA replication resulting in G:C→T: A transversion mutations [75–77] (Fig. 3). When UVB-induced CPDs and 6-4PPs are incorrectly resolved or UVA-induced oxidation lesions are not repaired timely, mutations including C: G → T: A transition, CC:GG → TT: AA tandem mutations or A: T → T: A transversion will form [78–80]. These mutations called “UV signature mutations” could be detected in a variety of skin cancers and are used to identify UV-induced DNA damage [66, 81]. While ROS can indeed damage DNA, an effect that should not be ignored is the oxidation of critical intracellular proteins including nucleotide excision repair (NER) proteins, thereby increasing the possibility of mutations in genetic materials caused by UVB [82–84]. Interestingly, reducing oxidative stress levels can prevent UV-induced cellular oxidative death and carcinogenesis [85, 86].

#### Mutation genes

Finally, these changes will accumulate over time, which could cause high-mutational background in skin cells and overwhelms DNA repair mechanisms and further potentially contribute to the emergence as well as development of skin cancer [71, 87, 88]. Malignant melanoma, resulting from the intricate interaction of developmental signals and genetic events, is further aggravated by UV radiation [89–91]. Exposed to UV light, the MC1R-PTEN axis in melanocytes is considered an important response center. MC1R has been found to promote the repair of UV-induced DNA damage whereas MC1R variants exhibit defects in their interaction with PTEN upon UV exposure so that they fail to inhibit the PI3K/ AKT signaling pathway driving the carcinogenic transformation of melanoma [92]. In addition, homeostatic processes can be disturbed by some genetic events after UV exposure. Melanomagenesis can occur as a result of melanocyte stem cells (MCSCs) proliferation and migration to the epidermis where they differentiate into melanocytes mediated by the overexpression of High Mobility Group AT-Hook 2 (*HMGA2*) after UVB exposure [93]. Beyond that, *BRAF*, *NRAS*, and *CDKN2A* are the major mutation genes related to the occurrence of melanoma [94–96]. *TP53/Trp53* gene frequently subjected to mutational events in skin carcinogenesis is hypothesized to constitute an early molecular vulnerability in the UV-radiation-driven carcinogenic cascade [65, 88, 97, 98]. It has been found that the UV-induced mutations of *TP53* or *PTCH* where “UV signature mutations” usually exist have a potential acceleration of BRAF(V600E)-driven melanomagenesis and are strongly associated with the development of BCC [46, 99–103]. Keratinocytes carrying *TP53* mutations can gain a growth advantage by resisting apoptosis, which allows them to eventually give rise to AK and SCC by clonal amplification. Sunlight-induced production of CPDs and the resulting decrease in DNA repair proficiency conspire to significantly modulate the *TP53* mutation spectrum observed in skin malignancies [101, 104, 105]. Analysis of the mutational landscape across 13 oncogenic genes within a cohort of 57 BCCs revealed a high frequency of genetic alterations. Predominant mutations observed in *PTCH1* and *TP53* genes that were mutated in 71.9% and 45.6% of the BCCs respectively consistent with UV-induced fingerprint mutations characterized by C > T and CC > TT transitions [106]. Simultaneously, a multitude of well-documented melanoma driver mutations and other frequently observed mutations are found at sites of UV-induced CPDs in melanocytes [107]. According to a recent study by Gabriel *et al.*, UV radiation facilitates blastic plasmacytoid dendritic cell neoplasm (BPDCN) by allowing plasmacytoid dendritic cells (pDCs) carrying potential pathogenic genes *TET2* to acquire additional genetic mutations. Concurrently, BPDCN tumor cells or precancerous cells in patient skin significantly enrich for genetic mutation signatures associated with UV exposure [108].

### Immunosuppression

#### Launch immunosuppressive molecular events

The immunosuppressive effects of UV exposure begin with early molecular events that induce downstream biological effects. The chromophores mainly including trans-UCA(urocanic acid) in the stratum corneum and nucleotide bases in epidermal keratinocytes or immune cells (such as Langerhans cells) can absorb energy and induce the corresponding cellular damage pathway when exposed to UV light [109]. After UVB reaches the skin, platelet-activating factors (PAFs) and platelet-activating factor receptor (PAF-R) agonists can generate to bind with PAF-R for the generation of UVB-mediated ROS and subcellular microvesicle particles (MVP) carrying the mediators with the ability of mediating systemic immunosuppression and thereby enhancing tumor growth are released [110]. Mast cells can be called from the skin to the draining lymph nodes by chemokine signaling due to PAF activation of their epigenetic alterations, where they secrete immunogenic cytokines and inhibit germinal center formation [111]. CPDs still play an important role in a series of cellular and molecular events related to immunosuppression. The reduction of CPDs in the epidermis could prevent the suppression of delayed and contact hypersensitivity reactions and systemic immunity caused by UV light [112]. The immunosuppressive mechanism of other molecule such as Tryptophan and 7-dehydrocholesterol has also been gradually clarified [109, 113, 114].

#### Downregulate the functionality of immune cells

In addition, UV exposure can suppress immune cell functions including immune response, induce them to exhibit an immunosuppressive state and expansion of regulatory T cells (Treg). Excessive UV irradiation, based on molecular perturbations and genetic lesions, impairs the homeostatic functionality of cutaneous immune cells. This includes a diminution in the phagocytic and chemotactic proficiency of neutrophils, alongside a decrement in their adhesive properties, an escalation in the apoptosis of peripheral blood mononuclear cells (PBMCs), and a marked attenuation of the antigen-presenting efficacy of human Langerhans and dermal dendritic cells (DCs) [115, 116]. UV light exposure has been shown to dysregulate T cell migration patterns by interfering with Sphingosine-1-phosphate (S1P) signaling and causes systemic immunosuppression contributing to the development of skin cancer [117]. Following UVB irradiation, CD11b^+^Langerin^-^ DCs manifest an upregulation of CD86 expression accompanied by an enhancement of tolerogenic gene transcription linked to the functionality and proliferative capacity of Tregs [118]. UVB exposure also induces the skin infiltration of Gr-1^+^CD11b^+^ myeloid cell often referred to as myeloid-derived suppressor cells (MDSCs) that usually play an immunosuppressive role in inflammation and tumor microenvironment [119].

#### Stimulate the production of immunosuppressive factors

Meanwhile, production of factors with immunosuppressive effects can also be promoted by UV exposure. Chronic exposure to UV radiation induces the activation of select skin immune cell subsets such as retinoic acid receptor-related orphan receptor gamma t (RORγt)–differentiated innate lymphoid cells (ILCs) and T lymphocytes, resulting in a perturbation of local cytokine landscape characterized by an enrichment of IL-22 and IL-17A associated with the proliferation of mutated keratinocytes (KCs) [120]. A variety of mediators with immunosuppressive effects such as PGE_2_, cytokines including IL-6, 8, 10 and tumor necrosis factor-alpha (TNF-α) could be produced when PAFs and PAF-like ligands bind with PAF-R and induce downstream immunosuppressive biological effects [111].

### Inflammation

#### Production of inflammatory mediators

Chronic inflammation is acknowledged as a pivotal factor that propels the transformation of epidermal cells and their progression towards malignancy [121, 122]. Prolonged or excessive exposure to UV radiation can lead to a state of chronic inflammation in the skin. This is due to the induction of DNA damage and alterations in the ECM by UV [123, 124]. Following UV radiation, many inflammatory mediators (such as inflammation-associated cytokines, chemokines, prostanoid-synthesizing enzyme COX-2 and lipid mediators) are released from cells residing in the skin, which is likely to be mediated by the UV-induced release of self-RNAs as a damage-associated molecular pattern dependent on Toll-like receptor 3 (TLR3) and Toll-like receptor adaptor molecule 1 (TRIF) from keratinocytes [104, 125, 126]. These mediators can activate the STAT3 and NF-κB signaling pathways closely related to carcinogenesis and development, thereby promoting tumor proliferation and anti-apoptotic capabilities [127–129]. Besides, the activation of the TGFβ1/SMAD3 signaling cascade by chronic UV exposure instigates photo-inflammation processes and bolsters malignant biological behaviors of skin cancer cells [130].

#### Activation of inflammasomes

UVB can also disrupt protein synthesis and induce DNA damage within cells, causing ribosomes to collide with other molecules in the cytoplasm and producing ribotoxic stress response (RSR), which initiates an activation of the NLRP1 inflammasomes facilitating the maturation and subsequent excretion of key pro-inflammatory cytokines (such as IL-1β, IL-18) that promote the development of skin cancer by enhancing inflammation and immune suppression [131, 132]. Further, long-term chronic NLRP1-dependent inflammation may increase a patient's risk of developing SCCs [133, 134].

Interestingly, male mice exhibited greater sensitivity to UV exposure, manifesting more severe inflammatory damages and an earlier onset of skin cancers [135]. This phenomenon suggested that gender may influence carcinogenesis due to different levels of UV-induced inflammatory response. From the different aspects, suppressing UV-induced inflammation was also accompanied by suppressing skin photocarcinogenesis [136]. Nonsteroidal anti-inflammatory drugs such as aspirin(ASA) are capable of delaying SCC onset induced by chronic UVB [11]. Genetic ablation of TNF-α in transgenic mouse models attenuated the progression of SCC subsequent to repetitive UVR treatment [137]. Collectively, these findings highlight the pivotal role of UV-induced inflammation in cancer development, emphasizing the need for further research to elucidate its mechanisms and potential therapeutic targets.

### Epigenetic alterations

Cancer initiation and progression are caused not solely by UV-induced mutations in significant genes but also by the subsequent alterations in epigenetics, which result in their abnormal expression patterns conducive to cancer development and aggressive behavior. Increasing research has found accumulation of epigenetic changes can drive cancer development as well as progression and alterations in the epigenome are capable of inducing the emergence of cancer apart from genetic mutations [138–140]. Under constant exposure of UV radiation, the cutaneous layer demonstrates a propensity for epigenetic shifts, such as the methylation of genomic DNA, histone acetylation and the posttranscriptional governance of noncoding RNAs, which leads to skin cancer [141–148].

#### Methylated modification

UV radiation-induced alterations in DNA methylation encompasses both hypomethylation and hypermethylation affecting genomic stability, which could lead to the silencing of tumor suppressor genes or the upregulation of oncogenes [146]. The methylation signatures observed in AK exhibit canonical characteristics of oncogenic methylation patterns and demonstrate a certain resemblance to the epigenetic profiles of cSCC, which mainly showed typical features of stem cell methylomes [149]. Meanwhile, analysis of transcriptomic and epigenomic changes during different stages of UVB-induced skin cancer has found that differentially DNA methylated regions mostly located in the distal intergenic regions and the promoters varied over time. These changes in methylation levels were associated with different stages of skin cancer development and were more pronounced in the early stages [150]. Epigenetic modification of cytosine can also promote the formation of UV-indued CPDs, resulting in mutagenesis effects. For instance, cytosine methylation promotes increase of UVB-induced CPD formation nearly 2-fold relative to the unmethylated [151]. The highest levels of CPDs produced under UVB radiation near sequences containing 5-carboxylcytosine (5caC) produced by DNA methyltransferases and 5mC oxidases at CpG dinucleotides [152].

#### Histone modification

UV-induced epigenetic carcinogenesis also involves the regulation of chromatin structure through histone changes. Yao *et al.* found UV decreased global H3K27ac characterized as a crucial epigenetic hallmark for delineating super enhancer (SE) landscapes across promoter regions and genome-wide remodeling of H3K27ac distribution in response to increasing UV exposure was observed. Notably, an enrichment of genes linked to UV-elicited SEs was detected within cancer-related and DNA damage response pathways [141]. UV damage leads to rapid and significant decondensation of heterochromatin, primarily mediated by DNA damage binding protein 2 (DDB2), which facilitates the displacement of linker histones from chromatin. This instability of heterochromatin structure is closely associated with cancer development and could be partly repaired by histone methyltransferase SETDB1 that is specifically recruited to the UV-damaged heterochromatic regions and maintains the H3K9me3 modification on newly deposited H3 histones, helping to protect the cell from the effects of genomic instability [153].

#### Noncoding RNAs modification

In addition, the carcinogenic effects of UV radiation are mediated by epigenetic modifications that extend beyond the DNA to encompass RNA as well. Upon UVA radiation, melanocytes emit extracellular vesicles laden with diverse cellular components notably miR-21 mediating the signal transduction in the keratinocytes and leading to their increased proliferation and heightened anti-apoptotic mechanisms, which links to the development of skin cancer [154].UVB irradiation could reduce the expression of miR-137 that could inhibit the tumorigenic phenotype in human epidermal melanocyte cell and increase the expression of miR-29a-3p and miR-183-5p that could downregulate the phosphatase and tensin homolog (PTEN) involved in the metastatic progression of cSCC [155–157]. In the latest research findings, the m^6^A methylation modification mediated by the methyltransferase METTL14 can regulate the maturation process of miRNAs under UVB exposure, thereby controlling the photo-aging of skin fibroblasts [158]. Further, UV radiation triggers a miRNA signature in primary SCC cells that mirrors aspects of the metastatic phenotype, implying a role for UV-induced epigenetic modifications in both cancer initiation and subsequent metastatic spread [157].

As mentioned above, epigenetic modifications can induce the upregulation of chemokine genes, thereby facilitating mast cell migration to induce immunosuppression [159, 160]. However, epigenetic changes are reversible and can be reversed. Phytochemicals such as epigallocatechin gallate (EGCG) have shown potential in preventing the malignant conversion of UV-induced papilloma into carcinoma or reactivating tumor suppressor genes that have been epigenetically silenced by reversing UV-induced epigenetic modifications for its function in suppressing DNA methyltransferases (DNMTs) [161–165]. These findings suggest that epigenetic alterations could potentially serve as a viable target in strategies aimed at preventing UV-mediated carcinogenesis.

## The role of UV in cancer treatment

**Fig. 4:**
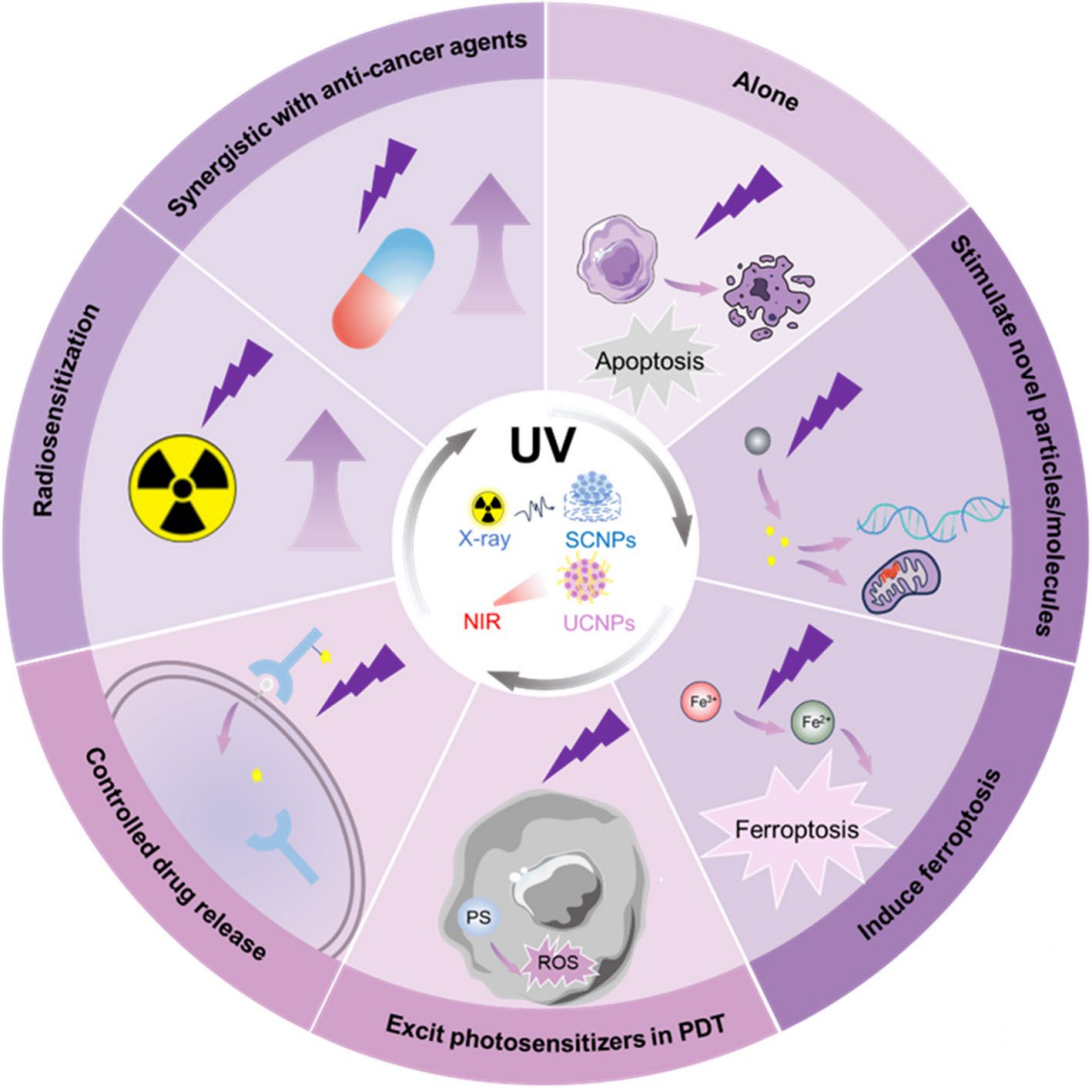
Summary of UV in cancer treatment. UV light can be generated by X-rays and near-infrared light through the excitation of scintillating nanoparticles (SCNPs) and upconversion nanoparticles (UCNPs), respectively, thereby circumventing their poor penetration drawbacks. In addition to its inherent ability to induce apoptosis in tumor cells, UV light can also be combined with nanomaterials to achieve various therapeutic effects in cancer treatment. These effects include synergistic actions with anti-cancer agents, radiosensitization, excitation of photosensitizers in photodynamic therapy, controlled drug release, induction of ferroptosis, and activation of novel particles or molecules

While previous research has primarily focused on the intricate mechanisms underpinning UV-induced carcinogenesis and progression, UV light remains a paradoxical force (Fig. 2). This dual nature is particularly reflected in contemporary research where nanomaterials are extensively explored and UV-light is often harnessed as an activating energy source to eradicate cancer cells. Below, we delve into some of the prevalent scenarios where UV light finds widespread application in cancer therapy today (Fig. 4).

### UV alone

UV alone can induce apoptosis and inhibit growth of a variety of tumor cells through different pathways depending on wave length, dose and cell lines with UVC irradiation at the highest frequency [166]. This effect has been observed in vitro in various tumor cell lines including human osteosarcoma cells, fibrosarcoma cells, glioblastoma cells, pancreatic cancer cells and colon cancer cells with EGFR signaling pathway being of great significance [166–172]. One possible mechanism is that UV damages DNA and induces cell cycle arrest followed by apoptosis [167]. Moreover, UVC radiation can affect the growth and spread of tumors by regulating cell-to-cell interactions in the tumor microenvironment. For instance, exposure to UVC radiation stimulates the secretion of many cytokines including TNF-α and IFN-γ of bone marrow-derived mesenchymal stromal cells (BM-MSCs), which collectively exert an inhibitory effect on the growth capacity of colorectal cancer (CRC) cells and induce their apoptosis [173]. The use of UVC in vivo experiments in cancer therapy has also been explored. UVC exposure not only suppressed cancer cells growth in nude mice with minimal residual cancer (MRC) but also appeared to be devoid of any significant side effects, which means the potential use of UVC following surgical resection in patients with MRC. Moreover, UVC exhibits greater efficacy in inhibiting cancer cell proliferation in 2D monolayer cultures, whereas UVB is more effective in reducing cancer growth in a skin-flap mouse model implanted with tumor cells. Additionally, in an experimental brain metastasis model using Lewis lung carcinoma (LLC), UVC irradiation was delivered through a craniotomy open window successfully and induced apoptosis in cancer cells. This UVC irradiation was particularly effective against LLC and significantly prolonged the survival of mice with brain metastasis. In clinical trials, near-ultraviolet light (NUV) has been used for nerve visualization in surgery and shown beneficial effects within the surgical field [174]. Clinically, similar to the carcinogenesis mechanism discussed above, UV alone plays a role mainly by inducing T cell apoptosis and regulating immune and inflammatory responses as a kind of physiotherapy for cutaneous T-cell lymphomas (CTCL), which is a relatively superficial skin tumor. The results obtained emphasize the capacity of UV light to combat cancer, specifically in the eradication of superficial tumors, thus introducing a prospective strategy in cancer management [166, 175, 176].

### Synergistic with anti-cancer agents

Further, UVC combined with anti-cancer agents may produce synergistic effects. Kawaguchi *et al.* reported that small dosages of UVC and cisplatin together have a synergistic against cancer impact by suppressing receptor tyrosine kinases (RTK), offering a unique therapy option for attempts to treat colorectal cancer [177]. Chang *et al.* has undertaken an extensive exploration in this field. Their team reported that the combined treatment of anti-oral cancer natural products with UVC radiation has enhanced the inhibition of cells proliferation compared to individual treatments alone and promoted apoptosis of oral cancer cells by enhancing ROS generation, cell cycle disturbance, mitochondrial dysfunction and DNA damage [178–184]. The relevant contents are summarized in Table 1. In general, UVC has a certain clinical translational application prospect in combined therapy [185].

**Table 1:** Summary of UVC alone or in combination with agents/natural products for tumor therapy in vivo and in vitro

Type of cancer/cell lines	Vitro/Vivo	Methods/Dose	Combined agents/Alone	Synergistically/lonely anti-cancer effect remarks	Mechanism	Ref
Human colon cancer SW480, HT29, DLD-1, HCT116	In vitro	5 s 10 J/m^2^	Cisplatin	Inhibited colony formation and induced apoptosis	Cell cycle disturbance; Downregulated receptor tyrosine kinases, such as EGFR and HER2	[177]
Salivary adenoid cystic carcinoma SACC-83/SACC-LM	In vitro	—— 10 J/ m^2^	Nimotuzumab	Enhanced UVC-induced apoptosis and inhibition of colony formation; Increased apoptosis	Reversed the EMT; Downregulated p-EGFR/p-Akt;	[186]
Human lymphoblastoid TK6	In vitro	20 min 1 mW /cm^2^	Ag NPs	Increased cytotoxic and genotoxic effects;	*H2AX* gene expression increased	[187]
Oral cancer Ca9-22	In vitro	1 J/ m^2^/s 30 J/ m^2^	CHW09	Radiosensitizer to UVC; Increased proliferation inhibition and apoptosis	Oxidative stress; DNA damage	[179]
Oral cancer HSC-3 and OC-2	In vitro	10 J/ m^2^ ——	CPC	Increased proliferation inhibition and apoptosis	Oxidative stress; DNA damage; Cell cycle disturbance; Mitochondrial dysfunction	[181]
Oral cancer Ca9-22 and CAL 27	In vitro	2 J/ m^2^/s 8 J/m^2^	PHA	Increased proliferation inhibition and apoptosis	Oxidative stress; DNA damage; Cell cycle disturbance; Mitochondrial dysfunction	[184]
Oral cancer Ca9-22	In vitro	2 J/m^2^/s 12 J/m^2^	EANA	Radiosensitizer to UVC; Increased proliferation inhibition and apoptosis	Oxidative stress; DNA damage; Cell cycle disturbance; Mitochondrial dysfunction	[182]
Oral cancer Ca9-22 and CAL 27	In vitro	1 J/m^2^/s 10 or 15 J/m^2^	FN	Increased proliferation inhibition and apoptosis	Oxidative stress; DNA damage; Cell cycle disturbance; Mitochondrial dysfunction	[183]
Oral cancer Ca9-22	In vitro	1 J/m^2^/s 14 J/ m^2^	MECCrt	UVC selectively killed oral cancer; Decreased cell viability; Increased apoptosis	Oxidative stress; DNA damage; Cell cycle disturbance; Mitochondrial dysfunction	[178]
Human pancreatic cancer Panc1, MiaPaca2, KP3 and BxPC3	In vitro	200 J–500 J	Alone	Proliferation inhibition; Induced apoptosis	Downregulated EGFR; Apoptosis via mitochondrial pathway	[168, 169]
Human colon cancer SW480, HT29, DLD-1	In vitro	20 s 10–200 J/ m^2^	Alone	Inhibited proliferation	Downregulated EGFR; Cell cycle disturbance;	[170]
Human colon cancer HT-29, SW1116, and SW620	In vitro	10 J/m^2^	Alone	Proliferation inhibition; Induced apoptosis	By BM-MSCs secreting cytokines like TNF-α, IFN-γ; Suppression of PI3K/AKT signal pathway	[173]
Model: minimal residual cancer (MRC) with athymic NCR nude (nu/nu) mice injected LLC-dual-color cells (Lewis lung carcinoma)	In vivo	Reopened incisions and UVC was irradiated 370mW/cm^2^ 100 J/ m^2^	Alone	Suppressed tumor growth in all treated mice; No apparent side effects of UVC exposure were observed	——	[166]
Model: Superficial Brain Tumors (Human glioma cell U87) and Metastasis (Lewis lung carcinoma, LLC)	In vivo	Beamed through the craniotomy open window: 370mW/cm^2^ 100 J/ m^2^	Alone	Decreased tumor size and extended survival of the mice with experimental brain metastasis (LLC), Glioma(U87) relatively resistant	Induced apoptosis;	[175]

*Abbreviations*: *Ag NPs* silver nanoparticles, *CHW09* Sulfonyl Chromen-4-Ones, *CPC* Cryptocaryone, *PHA* Physapruin A, *EANA* Ethyl acetate extract of Nepenthes adrianii × clipeata. *FN* Fucoidan, *MECCrt* methanolic extracts of Cryptocarya concinna roots

### Radiosensitization

Many tumors exhibit radiotherapy resistance due to their hypoxic characteristics so enhancing radiotherapy sensitivity has become an urgent problem [188, 189]. Unlike X-ray, UVC photons can damage genetic material without much reliance on oxygen, thereby enhancing radiotherapy sensitivity in tumors with hypoxic characteristics [190]. Interestingly, scintillating nanoparticles (SCNPs) namely low-conversion nanoparticles with the ability to absorb X-rays and convert them into lower energetic UVC photons have been explored in radiation therapy. Due to the substantially higher X-ray absorption cross-sections of inner-shell electrons relative to those of outer-shell electrons, elements with high atomic numbers, particularly transition metals and heavy metals which possess a significant number of inner-shell electrons, are promising candidates for the development of nanoscintillators. Lutetium phosphate doped lanthanide elements such as Tb^3+^ or Ce^3+^, for example LuPO4:Pr^3+^, are the very ideal material with high values of atomic number, emission intensity and appropriate emission spectrum [191]. Müller *et al.* has reported that combining LuPO4:Pr^3+^ with the ability to convert X-rays into UVC radiation due to the inter-configurational 4*f*^1^5*d*^1^-4*f* transition in Pr^3+^ with traditional X-ray treatment could result in a significant viability loss in hypoxic tumor areas compared to radiation alone and a significant formation of UV-specific DNA damage namely CPDs can be detected. This novel material can utilize UV radiation to reduce the ability of tumor cells to resist radiation [192]. At the same time, it helps to reduce the radiation dose while maintaining the same tumor control rate from the perspective of radiotherapy [191, 193].

### Excit photosensitizers in PDT

Harnessing the relatively high energy of UV light, researchers have explored its applications in combination with materials to activate photosensitizers to generate ROS in PDT that triggers downstream effects upon most occasions. PDT possesses the characteristics of non-invasiveness, excellent tumor selectivity, easy operation and few side effects. The three primary elements of PDT are photosensitizer (PS), light, and tissue oxygen [194]. When the photosensitizers are ingested by tumor cells with high metabolic activity, ROS will be produced and cause tumor cell apoptosis, necrosis or autophagy after irradiation with an appropriate wavelength light source [195, 196]. Although UV wavelengths match the absorption wavelength of many photosensitizers, it simultaneously poses several challenges for UV to excite materials directly for therapy as mentioned previously for prolonged UV exposure potentially causing DNA damage, immunosuppression, inflammation, photoaging and other harmful effects and finally leading to photocarcinogenesis. Moreover, the limited penetration depth of UV light restricts its reach to tissues at depths of approximately 100 μm [197]. Thus avoiding UV light's unsatisfactory penetration capacity while making use of its excitation property is a pressing concern. Intriguingly, scintillating nanoparticles and upconversion nanoparticles (UCNPs), which are typically activated by X-rays and near-infrared light (NIR) respectively, serve precisely this function. These two different forms of PDT excitation are called X-PDT and NIR-PDT respectively. These materials both avoid the low penetration of UV and take advantage of its excitation power in a transformational manner. The former has been described in the previous section. The latter has the ability to convert two or more low-energy pump photons from the near-infrared spectrum into a higher-energy UV photon with a shorter wavelength if it has been doped with appropriate type and amount of ions like Ce^3+^, which could better match the absorption band of the selected photosensitizers [198, 199]. In order to understand the conversion generation process of UV photons, it is also necessary to understand the structure of UCNPs. UCNPs typically incorporate dopant ions such as the activator like Tm^3+^ and sensitizers like Ce^3+^or Yb^3+^ within a host lattice commonly NaYF_4_ or NaGdF_4_, which are integral to the UCNPs' functionality. The activator ions are primarily responsible for the upconversion whereas sensitizers enhance the efficiency of this process. Upon NIR irradiation, sensitizers facilitate energy transfer to the activators, which promotes their excitation to a higher energy state and enables the emission of upconversion luminescence. This synergistic interaction between sensitizers and activators is essential for the realization of efficient upconversion luminescence in UCNPs [200]. Upon UV light, compatible photosensitizers notably TiO_2_ are energized, triggering the promotion of electrons from their valence band to the conduction band, resulting in the formation of highly reactive electron-hole (e-/h+) pairs [201, 202]. These electron-hole pairs can subsequently engage in reduction or oxidation reactions with chemical species present on the TiO_2_ surface and lead to the production of various cytotoxic ROS such as superoxide anions, which exhibit potential for cancer cell eradication in PDT [203–206]. In parallel, Cu-Cy nanoparticles exhibited a pronounced generation of ROS predominantly in the form of singlet oxygen (^1^O_2_) offering compelling substantiation for their promising role as potent ROS generators capable of eliminating cancer cell [207, 208]. To sum up, the application of SCNPs and UCNPs has indirectly made it possible for UV to be used in PDT for treatment of deep cancers. Some typical X-PDT applications together with UV sensitization are summarized in Table 2.

**Table 2:** Summary of X-PDT and radiosensitization mediated by SCNPs

Type	Nanoscintillator	In vitro/In vivo	Energy or dosage of X-ray (In vivo In vitro)	Emission wavelength (nm)	Cancer cells	Effect	Ref
X-PDT	LiYF_4_:Ce^3+^@SiO_2_ @ZnO	In vivo In vitro	8 Gy 0,2,3,4,6 Gy	305/325	HeLa	ROS generation	[209]
	LiLuF_4_:Ce^3+^@SiO_2_@Ag_3_PO_4_@Pt (IV)	In vivo In vitro	4 Gy 0,4,5,10,15,20 Gy	305/325	HeLa	Cisplatin production; ROS generation	[210]
	LiLuF4:Ce^3+^@Mn_2_(CO)_10_	In vivo In vitro	6 Gy 0, 2, 4, 6, 8 Gy	306 -326	HeLa	Generation of CO and MnO_2_ for tumor inhibition; ROS generation	[211]
	LiLuF4:Ce^3+^@RBS (Roussin's black salt)	In vitro	6 Gy 6 Gy,50 kV/75 µA	306 -326	A549; BEL-7402; HUVECs	Generation of NO, O_2_^−^, ONOO − ;	[212]
	Y_2.99_Pr_0.01_Al_5_O_12_ @PpIX@FA	In vitro	1.48 keV	300–450	4T1; RM-1; TRAMP-C1/C2; B16-F1; PyMT-R221A	ROS generation	[213]
	LaF3:Ce^3+^	In vivo In vitro	30,50,80 keV 215 Gy	340	U-87MG; F98	Potent radiation dose-enhancement effect	[214]
Radiation sensitizers	LuPO4:Pr^3+^	In vitro	2,4 Gy	220–285	HFF1; XP17BE	DNA damage caused by local UVC photons	[190]
	LuPO4:Pr^3+^, Nd^3+^	In vitro	6,9 Gy	200–280	A549	DNA damage caused by local UVC photons	[215]

### Controlled drug release

Selective and efficacious drug delivery systems significantly augment the efficacy of cancer treatment strategies. However, the traditional chemotherapy drugs commonly used in clinical practice are not selective to target tissues and organs so that treatment may require large amounts of drugs with many side effects. A selective method of delivering medication to certain diseased tissues makes use of drug carrier systems, which are often based on materials on the nanoscale [216]. Nanocarriers are taken in by diseased tissues through passive or active targeting strategies in which process achieving a controlled release of therapeutic drugs at precise sites and times is imperative for maximizing the therapeutic impact while minimizing side effects [217]. Synthetic stimuli-responsive biomaterials can change its physical and chemical properties under internal and external stimuli to achieve controlled drug release. Among various stimulation types, light stimulation has attracted considerable interest due to its noninvasiveness and precise temporal and spatial control [218, 219]. UV light stands as the preeminent light source in photo-controlled drug delivery systems attributed to its ample energy that elicits structural transformations in a wide array of light-responsive materials, which mainly includes three types namely cleavage, isomerization, cross-linking/decross-linking reactions, and then therapeutic drugs are released [22, 217, 220]. Regarding the field of precision medicine, central to promising UV-controlled drug release lies the utilization of UV-responsive photoremovable protecting groups (PPGs) such as nitrobenzyl (NB), coumarin (CM), azobenzene (AZO), spiropyran (SP) derivatives [22, 218, 221, 222]. These moieties serve as bridges between the therapeutic payload and the carrier, ensuring that the drug remains inactive during circulation. The application of UV light to PPGs within the tumor microenvironment elicits sophisticated structural transformations, achieving the precisely timed and spatially controlled release of drug. Coumarin and cinnamates are capable of undergoing photo-induced cross-linking reactions through [2π + 2π]-cycloaddition when exposed to UV light while they undergo decross-linking reactions when irradiated with light of a shorter wavelength [223]. Similarly, the hydrophobic moiety SP can isomerize into the hydrophilic conformation of merocyanine (MC) by UV to break intramolecular carbon–oxygen bonds [224].

This property of UV light has been harnessed to facilitate its application in targeted cancer therapy. The application of UV photo-control technology in antibody-drug conjugates (ADC) achieves the harmonious unification of targeting effects and highly spatiotemporal controllability in drug release. Li *et al.* reported a novel ADC in which UV has been used to cleave o-nitrobenzyl structure in its linker. Upon exposure to UV light, the ADC swiftly liberated cytotoxins, effectively exerting profound cytotoxicity against drug-resistant tumor cells. More than that, the UV-activated ADC exhibited a targeting efficacy equivalent to the native antibody, coupled with enhanced stability and photoresponsive properties [23]. According to Wang *et al.*, coumarin-anchored low generation dendrimers were assembled. The coumarin substitutes were cross-linked with one another at 365 nm UV irradiation while the cross-linked assemblies broke down at 254 nm UV irradiation. This nanoparticle loaded 5-fluorouracil and a therapeutic gene encoding tumor necrosis factor-related apoptosis-inducing ligand (TRAIL) also demonstrated complementary anti-cancer efficacy under UV exposure [225].

### Induce ferroptosis

The exploitation of ferroptosis as a means to suppress tumor growth has garnered significant attention, surfacing as a burgeoning therapeutic approach for the management of treatment-resistant cancers [226–229]. It has been reported that UVB induces keratinocytes to accumulate oxygenated phospholipids specific to ferroptosis. Additionally, UVB causes a significant decrease in intracellular glutathione (GSH) levels and the expression of glutathione peroxidase 4 (GPX4), which fuels photoaging and contributes to keratinocyte death through ferroptosis [20, 230–232]. Interestingly, this process may be regulated by circular RNA and autophagy [233, 234]. Concurrently, the utilization of liproxstatin-1, an inhibitor of ferroptosis, has been effective in minimizing UV-induced cutaneous damage [235].

**Fig. 5:**
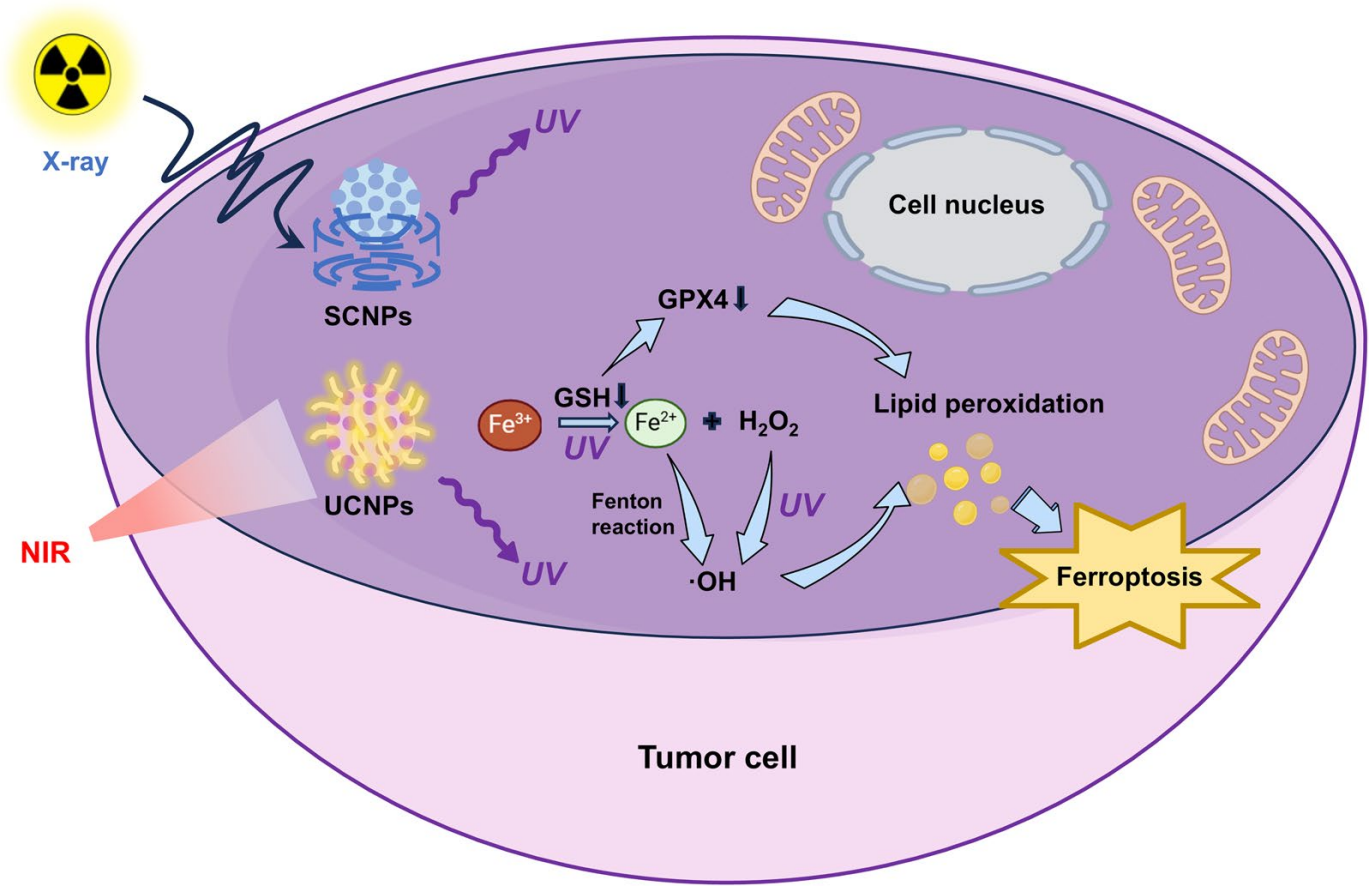
Summary of UV induced ferroptosis. During the PDT process, intracellular UV radiation can trigger ferroptosis. UV light emanating from excited SCNPs and UCNPs can reduce Fe^3+^ to Fe^2+^ within the cell, and then by Fenton reactions or directly catalyzing H_2_O_2_ into ·OH radicals, which lead to lipid peroxidation and finally ferroptosis. Additionally, the production of Fe^2+^ is accompanied by the depletion of GSH and a reduction in the activity of GPX4, which can further promote lipid peroxidation, ultimately leading to ferroptosis in cancer cells as well

Correspondingly, glioblastoma and breast cancer development could be inhibited by this property of UV radiation. When UV radiation from NIR excitation of UCNPs causes oxidative stress, GSH synthesis is consumed and inhibited, reducing the antioxidant capacity of the cells and encouraging lipid peroxidation events [236–239]. Besides, UV light prompts the reduction of intracellular Fe^3+^ to Fe^2+^, a catalyst for the Fenton reaction, which generates hydroxyl radicals (OH•) from H_2_O_2_ substrates and subsequently attacks the lipids of the cell membrane and initiates lipid peroxidation. In addition, the UV light from UCNPs can also directly photolyze H_2_O_2_ to produce •OH, increasing the generation of ROS and further promoting ferroptosis [240] (Fig. 5). Several animal experiments have further confirmed this result, showing that UV radiation emitted by the material induces ferroptosis and significantly inhibiting tumor growth as well as extending the survival time of animals [237–240].

### Stimulate novel particles or molecules

UV light could act as a high-energy stimulator to stimulate specific particles and molecules to eliminate cancer cells. Metal nanoparticles composed of multiple precious metals, which can selectively target cancer cells, exhibit a characteristic to be activated by ionizing radiation to generate high-energy photons that produce charged particles and free radicals in tissues and thus damage cancer cells. Nevertheless, conventional X-ray radiotherapy often causes irreparable damage to healthy cells along with cancer cells due to its high photon energy. UV light could be designed as a relatively safer approach by activating the nanoparticles within cancer cells without causing irreparable biological damage to normal tissues. When metal nanoparticles were irradiated with UV light in the wavelength range of 100-180 nm, the kinetic energy of the photoelectrons exceeded the minimum energy of 4.52 eV required for H-bond dissociation. Furthermore, irradiation with UV radiation in the range of 100-150 nm could achieve even higher energy levels of 6-7 eV that is sufficient to disrupt DNA double-strands and destroy cancer cell organelles. Notably, just one or two nanoparticles delivered to cancer cells are sufficient to generate numerous photoelectrons [241, 242]. Moreover, infiltrating the cancer cell membrane to facilitate targeted drug delivery and elicit anti-tumor responses remains a pivotal objective in cancer therapeutics. In 2017, García-López *et al.* used molecular machines (or molecular nanomachines) to drill through the lipid bilayers of cellular membranes and kill tumor cells by activating molecular motors with UV light to generate nanomechanical action. The UV light activated the smaller rotor part of the molecule, which together with the larger stator part formed this novel molecule. Additionally, this molecule can target tumor cells through specific cell surface recognition sites [24, 26, 27, 243].

However, it is necessary to conduct further animal-based studies to corroborate these results and ensure their efficacy and safety profile for future human applications. But in general, it holds promise for further development and application of UV radiation.

## Preclinical and clinical application and their limitations of UV radiation

UV light is a double-edged sword for cancer. UV light can cause skin cancer, while it can also synthesis some beneficial substances such as Vitamin D3 and can be used as an adjuvant therapy of treating tumor. In the early days, researchers often considered UV light a risk factor. Now UV light could also be used for phototherapy and play an auxiliary role in many aspects of tumor treatment.

For directly, UV therapy is a clinical treatment method used for cutaneous T-cell lymphomas (CTCL), especially for Mycosis Fungoides (MF) and Sézary Syndrome (SS) [244–246]. The majority of patients can experience remission from MF with phototherapy using UVA light in conjunction with 8-methoxypsoralen (PUVA) and narrow-band UVB light (NB-UVB). Extracorporeal photochemotherapy (ECP) works by exposing peripheral blood to 8-methoxypsoralen and UVA, and then re-infusing it back into the body to exert its therapeutic effect, which is also a kind of phototherapy method.

PUVA is primarily utilized for patients with deeper pigmentation and for higher-level phases [247, 248]. It is often combined with systemic active drugs and the most commonly used are interferon-alpha (IFN-α) and retinoids (isotretinoin). On a different note, NB-UVB therapy is primarily used to treat early MF and can be taken alone with a decreased risk [249]. However, for patients who are resistant to NB-UVB and have thick plaques, PUVA may be still essential [245]. However, there are few CTCL cases, and long-term reliable follow-up data are key to further determining the UV treatment methods. Simultaneously, it is essential to recognize that the restricted penetration of UV may be a key determinant in its restricted application, suggesting its suitability primarily for treating tumors confined to the body's surface. Considering the widely proven carcinogenic properties of UV, the evaluation of the safety of direct use of UV in the treatment of skin diseases and the risk of new skin cancers is very valuable. According to the retrospective risk assessment study and Meta-analysis of the use of UV for other diseases, which included a larger number of clinical patients, it was found that UV phototherapy did not increase the risk of skin cancer [250–252]. However, due to the limitations of the type of direct application of UV to tumor therapy, there have not been large-scale studies on the risks and safety of this type of phototherapy for skin tumors. Larger prospective studies and ongoing risk assessment in tumor will help further evaluate its safety. To enhance therapeutic benefits, more precise adjustments to therapy periods, dosage management, duration, and continuation would be needed.

For indirectly, UV radiation produced by excitation during PDT is used in the treatment areas of tumors mentioned above that, which type of tumor can be much deeper than direct-applied approaches. It must be considered that current research on generating UV for tumor treatment by exciting materials is still mainly focused on in vitro cell experiments and small animal models, and its potential for clinical translation, long-term safety, and effectiveness in larger animals or humans has not yet been verified [236]. Moreover, although small animal experiments have shown certain biocompatibility, the safety and potential side effects of using UV through these nanoparticles in the body over the long term still require further research [240]. Although several researches have evaluated the characteristics of in vivo metabolic kinetics, material clearance, tissue accumulation, and potential toxicity of materials used to induce UV, the boundary between the impact of different doses of nanoparticles on tumor growth through UV generation and the possibility of tissue damage also needs further demonstration [236, 238, 240].

As it is essential to consider that these nanoparticles did not cause significant damage to the normal tissues of mice, above all studies demonstrated acceptable biocompatibility and safety in cell or small animal models [236–240]. The fact that the excitation light source NIR is administered solely in the tumor region and that the nanoparticles generally have passive tumor targeting due to their enhanced permeability and retention effect suggests that there may not be as much of an impact on the surrounding normal tissues. Additionally, researchers have discovered that there is little to no heat produced when the material is excited by a laser [237, 240]. Although UV light is generally damaging to biological tissues, the UV light is generated internally within the nanoparticles from NIR light OR X-ray and this localized UV light production reduces the potential damage to surrounding normal tissues and avoid secondary carcinogenesis under the conditions as far as possible [239, 240].

Meanwhile, in order to take advantage of some of the characteristics of UV, PDT is often necessary. However, the significant risks and limitations associated with the therapeutic use of PDT have been appeared, most of which is mainly in the basic research stage. The existing clinical practice is to induce intracellular production of UV through PDT for the treatment of precancerous lesions and tumors. However, the clinical application of PDT is limited by light intensity, photoinitiators and tissue oxygen content [253]. For example, precancerous lesions and tumors of the oral mucosa are most often found on the tongue, palate, and buccal region. These areas are usually not effective for direct light sources thus diminishing the efficacy of PDT. Therefore, multiple phototherapy sessions are often required to minimize the risk of recurrence. In this process, PDT generates large amounts of ROS when it catalyzes the production of intracellular UV. Meanwhile, ROS induced by PDT may be toxic to normal cells, which may cause damage to normal tissue [254]. Photoinitiators also are the key factor for PDT effect to antitumor therapy. Traditional photoinitiators have the defects of poor water solubility and relatively poor initiation effect. Based on this, the researchers developed a series of novel photoinitiators such as UCNPs or SCNPs. However, as mentioned above, the degradation properties and biocompatibility of these nanomaterials need further consideration. Higher concentrations of nanomaterials can be significantly toxic to normal cells. What's more, PDT is dependent on the oxygen content of the tissue and requires oxygen as a supply to participate in ROS formation. However, the tumor microenvironment exhibits a state of hypoxia, which may be a limiting factor for PDT application. To solve these problems, researches developed to reconstruct the oxygen supplyment in the tumor microenvironment are needed.

UV radiation is most commonly used to trigger photocatalytic reactions, but the application of UV light is limited by low penetration depth. Apart from these, the followings should be unresolved in the future: Is it feasible to disrupt the traditional ROS pathway to prevent further exacerbation of hypoxia within the tumor microenvironment? Can adequate ROS be synthesized within the organism's reductive setting and directed towards DNA in tumor cells? How to accurately explain the process of photocatalytic therapy in vivo, and can the effects of photocatalytic materials be intuitively seen in vivo by means of in situ characterization? How can the toxicity of photocatalytic materials and the consumption of drugs due to metabolism or immunity be controlled?

## Conclusion and perspectives

Materials science has revolutionized the narrative of UV light, shifting from a historical perspective of harm to a modern horizon of therapeutic promise in cancer. Among the spectrum of UV light, UVC and UVB stand out as the most promising candidate for directly cancer treatment applications whereas UVA often serves as a stimulating factor for cancer treatment in conjunction with other materials. As new materials continue to surface, the exploration of UV light's utility in cancer therapy is set to expand, unveiling new avenues for research and cancer therapy. Nonetheless, we still lack innovative research models connecting basic research and clinical application, developing translational medicine and promoting the transformation of achievements. Theoretically, these following unresolved issues seem worthy of further exploration:

Since UV can be used directly or indirectly in cancer treatment, more studies of the dose-response relationship between UV and tumor development may help clarify the boundary between prospect and risk. It is also worth exploring whether this kind of intracellular UV radiation has different effects on tumor development than that exposed to extracellular under other similar conditions. Most malignant tumors are located deep in the body rather than on the surface. Although NIR light can improve the treatment depth, it cannot accurately locate the tumor. Advancing the application of UV light in tumor therapies necessitates further exploration on overcoming the constraints on light penetration, achieving selective targeting of tumors with photosensitizers and optimizing photo-response efficiency, which demands precision from both the UV and material perspectives. Optimizing the performance of UV radiation also necessitates the delicate manipulation of its temporal and spatial characteristics. Precise and accurate control of its application to diseased areas while protecting surrounding healthy tissue is also one of the directions and challenges for future research. Exploration is needed on the precise delivery of nanomaterials and the wavelength as well as dose effects of UV light for the dual effect of UV on tumor.

It is likely to start with the precise control of the light source. Digitalization and computer-controlled technology may assist to tackle these problems by automating the setting of UV light exposure time and irradiation dosage. It utilizes an integrated camera system and software algorithms to identify the contours of lesions, accurately projecting therapeutic light onto the affected areas. Through programming, it can systematically vary the light intensity to improve the efficacy of phototherapy [255, 256]. In addition, the local implantable ionic wireless light delivery device can also achieve precise control of the light source, but the disadvantage is that it requires surgical implantation of the device [257]. For materials that are excited to produce UV radiation, their surfaces can be modified to target the tumor microenvironment or specific antigens on the tumor cell surface to improve their delivery accuracy [258]. The effectiveness of indirect UV treatment based on materials should also consider the biocompatibility of the "all-in-one" strategy and the factors such as the loading efficiency of phototherapy agents, the sequence of stimulus response release, and the complexity of the environment where the stimulus factors are placed. On the one hand, it is necessary to find more suitable phototherapy agents. On the other hand, it is not entirely dependent on the idea of "assembly", but more necessary to go into the photo-response mechanism and develop new photo-response materials. Besides, the introduction of in vivo imaging such as fluorescence in phototherapy or multi-modality medical imaging including MRI, CT, PET can also determine the location and morphology of tumor lesions and monitor the distribution of therapeutic agents to produce intracellular UV radiation, so as to realize the integration of diagnosis and treatment [259, 260].

In general, UV directly or indirectly, alone or combined materials show certain application prospects in tumor therapy in the current research. However, its wide application also faces many challenges. The type of tumor that can be treated directly with UV is limited and exploration of clinical translation of the indirect application about UV in tumor treatment based on materials is still shallow. Long-term safety data of above materials in vivo remains further study. In addition, the ethical implications of using UV radiation directly or indirectly in therapeutic contexts, particularly in terms of patient safety and informed consent, should be concerned. And the practical challenges of translating these therapies from research to clinical practice, such as the development of safe delivery systems, dose optimization, and patient-specific factors, should be adequately addressed, as they are often significant barriers to the successful implementation of new therapies from bench to bedside.

## Data Availability

Not applicable.
